# Health Utilities in Patients with Type 2 Diabetes in Taiwan

**DOI:** 10.3390/healthcare9121672

**Published:** 2021-12-03

**Authors:** Chia-Chia Chen, Jin-Hua Chen, Chien-Lung Chen, Tzu-Jung Lai, Yu Ko

**Affiliations:** 1 Department of Pharmacy, College of Pharmacy, Taipei Medical University, Taipei 11031, Taiwan; ischiachia@gmail.com; 2 Graduate Institute of Data Science, College of Management, Taipei Medical University, Taipei 11031, Taiwan; jh_chen@tmu.edu.tw; 3 Statistics Center, Office of Data Science, Taipei Medical University, Taipei 11031, Taiwan; 4 Division of Nephrology, Landseed International Hospital, Taoyuan 32449, Taiwan; chencl4922@gmail.com; 5 Center for Drug Evaluation, Taipei 11557, Taiwan; joann7909026@hotmail.com; 6 Research Center for Pharmacoeconomics, College of Pharmacy, Taipei Medical University, Taipei 11031, Taiwan

**Keywords:** health utilities, diabetes mellitus, DM complications, EQ-5D

## Abstract

We aimed to measure health utilities in patients with diabetes mellitus (DM) in Taiwan and to estimate the impact of common DM-related complications and adverse effects (AEs) on health utilities. The present study was a cross-sectional survey of DM patients at a metropolitan hospital. Respondents’ health-related quality of life (HRQoL) was assessed by the EQ-5D-5L, and ordinary least-squares (OLS) regression was used to estimate the impact of self-reported DM-related complications and AEs on health utilities after controlling for age, gender, and duration of DM. A total of 506 eligible adults with type 2 DM (T2DM) were enrolled. The EQ-5D index values in our study sample ranged from −0.13 to 1, with a mean ± standard deviation of 0.88 ± 0.20. As indicated by the negative regression coefficients, the presence of any complication or AE was associated with lower EQ-5D index values, and the greatest impact on the score was made by amputation (−0.276), followed by stroke (−0.211), and blindness (−0.203). In conclusion, the present study elicited health utilities in patients with T2DM in Taiwan using the EQ-5D-5L. These estimated utility decrements provided essential data for future DM cost–utility analyses that are needed as a result of the increasing prevalence and health expenditures of DM.

## 1. Introduction

According to the 2016 Global Report on Diabetes published by the World Health Organization, there were 422 million adults with diabetes mellitus (DM) around the world in 2014, a quadruple increase since 1980, and the overall global DM prevalence had increased from 4.7% in 1980 to 8.5% in 2014 [1]. In Taiwan, there are more than 2.2 million people living with DM [2], and the prevalence of DM has risen from 8.5% in 2005–2008 to 12.3% in 2013–2015 [3]. Moreover, there were an estimated 463 million adults with DM in 2019 globally, as reported by the International Diabetes Federation, and this figure may rise to 700 million by 2045 [4]. With the growth of the DM population, DM has become a heavy burden not only to individuals but also to society as a whole.

Patients with DM have a lower health-related quality of life (HRQoL) than those without DM [5]. Previous studies found that DM-related complications could be key contributors to lower HRQoL [6,7]. Indeed, patients’ HRQoL worsens as complications arise and progress [7]. A few studies have measured utility decrements to estimate the impact of DM-related complications on HRQoL. A review by Beaudet et al. [8] summarized health utilities associated with DM-related complications and adverse effects (AEs) in studies that met the National Institute for Health and Care Excellence reference case criteria (i.e., studies that used the EQ-5D for the measurement of HRQoL). A total of 21 studies were included in the review, and the sample size of the included studies ranged from 17 to 4641. In the review, Beaudet et al. reported that the utility decrements associated with DM-related complications and AEs were: −0.014 for hypoglycemia, −0.055 for myocardial infarction (MI), −0.090 for ischemic heart disease (IHD), −0.108 for congestive heart failure (CHF), −0.164 for stroke, −0.164 for end-stage renal disease (ESRD), and −0.280 for amputation.

Given the rising prevalence and increasing expenditures of DM, this disease has become an important public health issue worldwide. How to ensure fair, reasonable, and cost-effective resource allocation among various treatment options for DM with limited resources is a top priority for every country. As a result, pharmacoeconomic evaluations, particularly cost–utility analysis (CUA) that compares both costs and health effects between different treatment options, can assist healthcare decision making for DM management. In a CUA, health effects are commonly expressed as quality adjusted life years (QALYs), which combine both the quality (i.e., the utility value of a health state) and quantity of life into a single index. Therefore, health utilities are an essential component for the calculation of QALYs in CUA studies. At present, DM-related utility data remains limited in Asia, let alone Taiwan. The present study aimed to estimate the health utilities associated with DM-related complications and AEs by using EQ-5D in patients with DM in Taiwan. The results provide essential data for future DM cost–utility analyses.

## 2. Methods

### 2.1. Study Design

The present study was a cross-sectional survey that examined health utilities using the EQ-5D. In addition, the survey questionnaire administered also collected HRQoL and DM-related information in order to estimate the impact of DM-related complications and AEs on health utilities in patients with DM in Taiwan.

### 2.2. Subjects

A convenience sample of outpatients with DM was recruited at a metropolitan hospital, mainly in the endocrinology and metabolism clinic, the nephrology clinic, and the dialysis center. In order to have a suitable number of patients with each complication and AE, we endeavored to recruit patients with at least one complication or AE of interest. As a result, the proportion of respondents with a particular DM-related complication or AE would be higher than that of the whole group of DM outpatients in the study hospital. The study hospital is a metropolitan hospital that provides comprehensive health care to the people of Taoyuan, which is a special municipality in northern Taiwan with a population of 2.25 million residents, making it the fifth-largest city in Taiwan. Currently, the hospital employs over 1000 workers, has 630 beds, and serves over 2500 outpatients daily. Subjects were included in the present study if they had a confirmed diagnosis of DM and were age 20 years or older. The exclusion criteria were: (1) under the age of 20; (2) currently pregnant; (3) cognitively impaired or unable to communicate with the survey administrators.

### 2.3. Study Questionnaire

The aim of the present study was to estimate the impact of self-reported DM-related complications and AEs on health utilities in patients with DM. The drafting of the questionnaire was in accordance with previous studies of health utilities and pharmacoeconomics in DM [9,10,11,12,13,14,15,16,17,18,19,20,21,22,23,24]. In addition, the original questionnaires that were used in two similar studies, one by Solli et al. [10] in Norway and the other one by O’Reilly et al. [11] in Canada, were obtained from the authors and used as references for the development of our study questionnaire.

The drafted questionnaire in the present study was reviewed by an endocrinology and metabolism specialist serving as the director of the division of Endocrinology and Metabolism, a DM educator, and a senior pharmacist at the study hospital. In addition, a pilot test of 25 eligible patients was performed at the beginning of the survey to improve the questionnaire’s readability and clarity. The final version of the study questionnaire consisted of two parts: background information and the EQ-5D. No change was required based on the feedback of the pilot testers.

The background information collected included respondents’ demographic characteristics (age and gender) and DM-related information, which included duration of DM, type of DM, and current DM treatment (e.g., lifestyle management, oral antidiabetic agents, insulin, and injectable non-insulin drugs). Patients were also asked to report DM-related complications and AEs.

The specific DM-related complications and AEs under examination included the following: symptomatic hypoglycemia, urinary tract infection (UTI)/genital infection, diabetic ketoacidosis (DKA), angina pectoris, diabetic foot ulcer, stroke, blindness, amputation, MI, CHF, and ESRD. As the medical term “ESRD” might have been too difficult for respondents to understand, we instead asked the respondents whether they had previously had dialysis. In addition, in the analysis, MI and angina pectoris were combined into one item labeled IHD. Thus, there were a total of 10 DM-related clinical events under assessment.

As the impact of DM-related clinical events on health utilities may change over time, the duration of the effects of these clinical events on utility decrements was taken into consideration. For acute illnesses such as hypoglycemia, UTI/genital infection, and DKA, which usually run a relatively short course, respondents were asked if they had experienced these clinical events within the previous month. Other conditions, such as diabetic foot ulcers, may last for a prolonged time; however, since their impact is the greatest in the acute phase and respondents might have become used to the discomfort later on, only respondents who had suffered from diabetic foot ulcers in the previous month were counted. Patients who had angina in the previous month or MI in the previous year were considered to have IHD. For chronic diseases such as stroke, CHF and ESRD, or those that usually leave permanent damage such as blindness and amputation, respondents were asked if they had experienced these clinical events within the previous year. In addition, the respondents were asked whether they had any other diseases that might lower their HRQoL.

### 2.4. EQ-5D

Respondents’ HRQoL was assessed by the EQ-5D-5L in the present study. The EQ-5D, a standardized instrument to measure HRQoL, is widely used to examine utility decrements due to disease. The EQ-5D-5L consists of a descriptive system where respondents are asked to indicate their current health status in five dimensions: mobility, self-care, usual activities, pain/discomfort, and anxiety/depression. Each dimension has 5 levels: no problems, slight problems, moderate problems, severe problems, and extreme problems. These levels of each dimension are taken together to create a 5-digit number that describes respondents’ current health status. For example, “11111” indicates that a patient has no problem in any of the five dimensions, while 12345 signifies no problem with mobility, slight problems with self-care, moderate problems with usual activities, severe problems with pain/discomfort, and extreme problems with anxiety/depression. With the absence of an EQ-5D-5L value set for Taiwan, the 5-digit number was converted to an EQ-5D index value between –0.39 (had extreme problems in all EQ-5D dimensions) and 1 (perfect health) using the China value set [25]; a higher index score indicates a better health status. In order to examine the robustness of the results, all analyses were repeated on the EQ-5D index values calculated using the value set for Hong Kong [26].

### 2.5. Data Collection

The survey was conducted from February 2018 to May 2018. The study questionnaire was interviewer-administered and took approximately 10 to 15 min to complete. The survey was conducted in the outpatient clinics by well-trained interviewers who remained nearby to provide assistance and to answer respondents’ questions. In addition, supplementary survey aids, such as a simplified study protocol and a pictorial with written explanations of medical terms and common treatments for certain complications, were provided. In addition to the information collected by the study questionnaire, the respondents’ most recent HbA1c value was obtained from their hospital medical records. To ensure data accuracy, the respondents’ type of DM was initially recorded during the interview and then double-checked by reviewing their medical records.

### 2.6. Data Analysis

For descriptive statistics, means and standard deviations (SDs) were used for continuous variables and frequency distributions for categorical variables. ANOVA with Scheffe’s test was performed to test the hypothesis that a lower EQ-5D index value was associated with having more DM-related clinical events. Moreover, ordinary least-squares (OLS) regression was used to estimate the impact of each DM-related complication and adverse effect on respondents’ health utilities after controlling for their age, gender, and duration of DM. All statistical analyses were performed in SPSS 19 (IBM, Armonk, NY, USA) with a *p*-value < 0.05 considered to be statistically significant.

## 3. Results

A total of 507 eligible adults with DM were enrolled. As only one respondent reported having type 1 DM, to preserve homogeneity, we excluded that patient from all analyses, yielding a study sample of 506 respondents. Descriptive statistics regarding the respondents’ demographics, DM-related characteristics (i.e., duration of DM, most recent HbA1c value, and DM treatment), and self-reported DM-related complications or adverse effects, are shown in Table 1. Among the 506 respondents, 56.5% were male, and the mean age was 60.1 years (SD = 13.1). The mean duration of DM in the respondents was 10.1 years (SD = 8.3), and the average HbA1c was 7.9% (SD = 1.8). As shown in Table 1, oral antidiabetic agents were the most commonly reported treatment (88.7%) used to control blood glucose levels in the study sample, followed by lifestyle management (33.6%), insulin (28.3%), and injectable non-insulin drugs (1.6%).

The number and type of DM-related complications, or AE, are reported in Table 1. Symptomatic hypoglycemia was the most common condition, which was reported by 119 (23.5%) respondents, followed by UTI/genital infection (108, 21.3%), IHD (85, 16.8%), stroke (60, 11.9%), ESRD (48, 9.5%), diabetic foot ulcer (42, 8.3%), CHF (41, 8.1%), DKA (15, 3.0%), blindness (11, 2.2%), and amputation (7, 1.4%).

In each EQ-5D dimension, the respondents’ perceived levels of problem are summarized in Table 2. Of the 506 respondents surveyed, the most commonly perceived problems were in the pain/discomfort dimension, with 38.0% of respondents reporting having pain or discomfort. In contrast, only 9.9% of respondents reported problems in the self-care dimension.

The EQ-5D index values in our study sample ranged from −0.13 to 1, with a mean ± SD of 0.88 ± 0.20. The ANOVA result showed that patients with more DM-related complications or AEs had a lower EQ-5D index value (*p* < 0.001). In addition, post-hoc comparisons found that in patients without any complications/AEs, the mean EQ-5D index value (0.95, SD = 0.09) was significantly higher than in both those who reported having two complications/AEs and those with three or more complications/AEs (0.78 and 0.72, respectively; *p* < 0.001 for each). In addition, the mean EQ-5D index value (0.92, SD = 0.13) elicited from patients with one complication/AE was significantly higher than in those who reported having two complications/AEs and those with three or more complications/AEs (*p* < 0.001 for each).

Table 3 presents the results of the OLS regression analysis of the relationship between the EQ-5D index value and DM-related complications/AEs. Male gender was associated with higher EQ-5D index values (β = 0.033, *p* < 0.05), while increased age (β = −0.002, *p* = 0.001) and increased duration of DM (β = −0.003, *p* = 0.01) were related to lower EQ-5D index values. As indicated by the negative regression coefficients, the presence of any complication or AE was associated with lower EQ-5D index values, and the greatest impact on the score was made by amputation (−0.276), followed by stroke (−0.211) and blindness (−0.203).

All of the analyses mentioned above were based on EQ-5D index values calculated by the tariffs from China. To make a comparison, the analyses were repeated using the value sets of Hong Kong, but no important differences were found in the results between the two countries.

## 4. Discussions

This study is one of the few that reports utility decrements in DM patients in an Asian country with a relatively large sample while including multiple DM-related complications and AEs. The negative regression confirmed that the presence of complications and AEs was associated with lower EQ-5D index values and also indicated the relative impact of each individual complication or AE. Specifically, our results showed that the greatest impact was made by amputation, followed by stroke and blindness. Similar rankings can be found in previous large-sample surveys by Clarke et al. [9] (3192 participants) and Hayes et al. [27] (11,130 participants). In addition, despite slight differences in rankings, in the study by Lin et al. [28], the complications with the lowest preference scores, as assessed by the SG method, were also blindness, stroke, and amputation. Given the great impact of these three complications on both HRQoL and health expenditures, it is important for patients with DM to have regular screening for peripheral neuropathy, peripheral arterial disease, and diabetic retinopathy. Health care providers should also strive to help patients improve their DM control in an effort to prevent DM-related complications and AEs altogether.

It is noteworthy that in our study, the R^2^ was 27.8% in the regression analysis, which was higher than those reported in Clarke et al. [9] (7%) and O’Reilly et al. [11] (5.8%). Respondents in the present study were asked to report if they had any other diseases that might lower their HRQoL, and this adjustment in the analysis may have explained the relatively high R^2^. In addition, the difference could be explained by the fact that the present study included ten DM-related complications and AEs in the analysis while fewer complications were included in the studies by Clarke et al. (six complications) and O’Reilly et al. (four complications). As such, the results suggest that factors affecting health utilities in patients with DM are likely complex, and future research is needed to further assess the determinants of low health utilities.

Similar to other studies conducted in other Asian countries (i.e., Indonesia [29], Vietnam [30], and Iran [31]), the present study demonstrated that having a DM-related complication or AE is associated with a lower EQ-5D index value. As such, minimizing complications is critical to maintaining HRQoL among patients with DM. Moreover, it was found that in the five dimensions of the EQ-5D, the domain that was most commonly perceived to be a problem was pain/discomfort, a result which was also reported in previous studies [10,29,31]. Many DM-related complications may lead to pain and discomfort. Moreover, insulin injections and the AEs of blood sugar lowering agents (e.g., hypoglycemia, SGLT-2 inhibitor-induced UTI/genital infection, and metformin-induced gastrointestinal symptoms) may also be associated with pain and discomfort. Therefore, patients with DM should be educated to take medicine properly in order to keep their blood sugar under control and reduce the risk of developing complications.

Given that the prevalence and health expenditures of DM are rising, DM has become a heavy burden to both individuals and society. The present study measured health utilities in patients with DM in Taiwan using the EQ-5D-5L and estimated the impact of DM-related complications and AEs on health utilities. The research findings provide a set of Taiwan-specific DM utility data, which is valuable and essential for future economic evaluations of new DM treatment alternatives, which evaluations are necessary given limited resources and the high prices of new drugs. Moreover, the study findings suggest that health care providers should dedicate time and resources to patient education about preventing DM-related complications and taking medications correctly. As a further step, our recommendations for future research include: (1) calculating EQ-5D index values using the Taiwan EQ-5D-5L value set when available because these tariffs are more representative of individuals’ preferences in Taiwan, (2) reproducing the present study with a multi-center, large-sample survey to enhance the external validity of the study findings, and (3) conducting a longitudinal survey to assess within-patient changes in HRQoL due to clinical events.

There are limitations to the present study. First, all of the DM-related complications and AEs in the present study were self-reported. Respondents might have forgotten, ignored, or misunderstood what was being asked, which could have led to the underreporting or overreporting of the conditions of interest. Second, patients with severe complications might have been too ill to participate, and the respondents were relatively healthy since they were recruited from outpatient settings. In addition, the respondents were recruited from only a single hospital. As a result, the study sample may not be representative of all patients with diabetes, and the impact of DM-related complications and AEs on health utilities might not have been fully captured. Third, despite our efforts to recruit patients with less common complications, a few complications still had relatively few respondents (e.g., amputation and blindness). As such, the estimated utility decrements with these complications should be interpreted with caution. Moreover, given the limited number of respondents in certain complication/AE groups, severity levels of the complications and AEs were ignored. Lastly, with the absence of an EQ-5D-5L value set for Taiwan when the study was conducted, our analyses were based on EQ-5D index values calculated by the tariffs from China, where preferences could be different from Taiwan.

## 5. Conclusions

The present study elicited health utilities in patients with DM in Taiwan using the EQ-5D-5L. It was found that the presence of any self-reported DM-related complication or AE was associated with lower EQ-5D index values. The utility decrements were measured to estimate the impact of DM-related complications and AEs on health utility. The complication with the greatest impact was amputation, followed by stroke and blindness. These estimated utility decrements provided a set of Taiwan-specific DM utility data, which is essential for future DM cost–utility analyses that are needed given the increasing prevalence and health expenditures of DM in Taiwan. In addition, the findings might also provide decision-makers with evidence to implement more appropriate policies for maintaining HRQoL among patients with DM.

## Figures and Tables

**Table 1:** Sample characteristics (*n* = 506).

Variable	Mean (SD)
Age (years)	60.1 (13.1)
Duration of DM (years)	10.1 (8.3)
HbA1C (%)	7.9 (1.8)
EQ-5D Index value	0.88 (0.20)
Variable	*n* (%)
*Gender*	
Male	286 (56.5)
Female	220 (43.5)
*Education*	
Never attended school	29 (5.7)
Elementary school	101 (20.0)
Junior high school	110 (21.7)
High school	177 (35.0)
Bachelor	72 (14.2)
Master/PhD	17 (3.4)
*DM Treatment*	
Lifestyle management	170 (33.6)
Oral antidiabetic agents	449 (88.7)
Insulin	143 (28.3)
Injectable non-insulin drugs ^a^	8 (1.6)
*DM-related complications and AEs*	
Symptomatic hypoglycemia	119 (23.5)
Urinary tract infection/genital infection	108 (21.3)
Ischemic heart disease	85 (16.8)
Stroke	60 (11.9)
End-stage renal disease	48 (9.5)
Diabetic foot ulcer	42 (8.3)
Congestive heart failure	41 (8.1)
Diabetic ketoacidosis	15 (3.0)
Blindness	11 (2.2)
Amputation	7 (1.4)
*Number of DM-related complications or AEs*	
0	200 (39.5)
1	148 (29.2)
2	67 (13.2)
≥3	91 (18.0)

SD, standard deviation; DM, diabetes mellitus; AE, adverse effect. ^a^ glucagon-like peptide-1 (GLP-1) receptor agonists (i.e., exenatide and dulaglutide).

**Table 2:** Distribution of respondents’ perceived levels of problem in each EQ-5D dimension.

EQ-5D Dimensions	Level of Perceived Problem, *n* (%)									
	1		2		3		4		5	
Mobility	367	(72.5)	87	(17.2)	31	(6.1)	11	(2.2)	10	(2.0)
Self-care	456	(90.1)	31	(6.1)	4	(0.8)	3	(0.6)	12	(2.4)
Usual activities	409	(80.8)	56	(11.1)	21	(4.2)	12	(2.4)	8	(1.6)
Pain/discomfort	314	(62.1)	142	(28.1)	42	(8.3)	4	(0.8)	4	(0.8)
Anxiety/depression	337	(66.6)	122	(24.1)	29	(5.7)	11	(2.2)	7	(1.4)

Level 1, no problems; Level 2, slight problems; Level 3, moderate problems; Level 4, severe problems; Level 5, extreme problems.

**Table 3:** Ordinary least-squares regression analysis of the relationship between EQ-5D index value and DM-related complications or adverse effects.

Variable	Coefficient	SE	*p* value	95% CI
Constant	1.089	0.038	<0.001	(1.02; 1.16)
Male	0.033	0.015	0.034	(0.002; 0.06)
Age (years)	−0.002	0.001	0.001	(−0.003; −0.001)
Duration of DM (years)	−0.003	0.001	0.01	(−0.01; −0.001)
Symptomatic hypoglycemia ^a^	−0.009	0.018	0.60	(−0.05; −0.001)
Diabetic-ketoacidosis ^a^	−0.022	0.086	0.80	(−0.19; 0.15)
Urinary tract infection/genital infection ^a^	−0.044	0.034	0.20	(−0.11; 0.02)
Ischemic heart disease ^b^	−0.050	0.033	0.134	(−0.12; 0.02)
End-stage renal disease ^c^	−0.135	0.038	<0.001	(−0.21; −0.06)
Diabetic foot ulcer ^a^	−0.136	0.050	0.01	(−0.23; −0.04)
Congestive heart failure ^c^	−0.169	0.039	<0.001	(−0.25; −0.09)
Blindness ^c^	−0.203	0.085	0.02	(−0.37; −0.04)
Stroke ^c^	−0.211	0.054	<0.001	(−0.32; −0.10)
Amputation ^c^	−0.276	0.169	0.10	(−0.61; 0.06)
Others	−0.074	0.016	<0.001	(−0.11; −0.04)

R^2^ = 27.8%. SE, standard error; CI, confidence interval. ^a^ Previous month. ^b^ Had angina in previous month or myocardial infarction in previous year. ^c^ Previous year.

## Data Availability

The data presented in this study are available on request from the corresponding author.
